# Enhanced CAD Detection Using Novel Multi-Modal Learning: Integration of ECG, PCG, and Coupling Signals

**DOI:** 10.3390/bioengineering11111093

**Published:** 2024-10-30

**Authors:** Chengfa Sun, Xiaolei Liu, Changchun Liu, Xinpei Wang, Yuanyuan Liu, Shilong Zhao, Ming Zhang

**Affiliations:** 1 Department of Biomedical Engineering, School of Control Science and Engineering, Shandong University, Jinan 250061, China; 201920518@mail.sdu.edu.cn (C.S.); liuyy@sdu.edu.cn (Y.L.); zhaoshilong@mail.sdu.edu.cn (S.Z.); 2 Department of Electrical Automation Technology, Yantai Vocational College, Yantai 264670, China; lixinpu2005@163.com; 3 Huiyironggong Technology Co., Ltd., Jinan 250098, China; zhangmingzw@163.com

**Keywords:** CAD, coupling information, entropy, recurrence plot, CNN, feature selection

## Abstract

Early and highly precise detection is essential for delaying the progression of coronary artery disease (CAD). Previous methods primarily based on single-modal data inherently lack sufficient information that compromises detection precision. This paper proposes a novel multi-modal learning method aimed to enhance CAD detection by integrating ECG, PCG, and coupling signals. A novel coupling signal is initially generated by operating the deconvolution of ECG and PCG. Then, various entropy features are extracted from ECG, PCG, and its coupling signals, as well as recurrence deep features also encoded by integrating recurrence plots and a parallel-input 2-D CNN. After feature reduction and selection, final classification is performed by combining optimal multi-modal features and support vector machine. This method was validated on simultaneously recorded standard lead-II ECG and PCG signals from 199 subjects. The experimental results demonstrate that the proposed multi-modal method by integrating all signals achieved a notable enhancement in detection performance with best accuracy of 95.96%, notably outperforming results of single-modal and joint analysis with accuracies of 80.41%, 86.51%, 91.44%, and 90.42% using ECG, PCG, coupling signal, and joint ECG and PCG, respectively. This indicates that our multi-modal method provides more sufficient information for CAD detection, with the coupling information playing an important role in classification.

## 1. Introduction

Coronary heart disease (CAD), a major contributor of increased global mortality, is induced by coronary atherosclerosis [1]. This condition is characterized by the long-term accumulation of cholesterol and fatty deposits within arterial inner walls, leading to plaque formation and arterial narrowing. Symptoms of CAD include dizziness, fatigue, and myocardial ischemia. In severe cases, sudden rupture of plaques can lead to acute myocardial infarction or even death [2]. Due to the irreversible nature of CAD progression, early and precise diagnosis is crucial in delaying its advancement and reducing mortality [3]. Although coronary angiography [4] is the gold standard for CAD diagnosis, its invasive and costly nature limits its widespread use. Alternatively, electrocardiogram (ECG) and phonocardiography (PCG), both noninvasive and affordable techniques, are more suitable for routine CAD screening. However, cardiovascular complex activities limits the efficacy of single-modal data in CAD analysis. To mitigate the limitation, multi-modal learning techniques by integrating diverse data sources provide a more comprehensive understanding of cardiovascular states, thus attracting attention in various fields.

ECG records crucial information about the electrical activity of the heart. Severe CAD patients present notable alterations in ECG recordings, such as ST-wave elevation or depression and T-wave inversion, serving as distinctive indicators of myocardial ischemia caused by CAD. However, early or moderate CAD patients frequently remain asymptomatic, potentially increasing the risk of misdiagnosis [5]. On the other hand, PCG carries essential information on cardiac mechanical activity. Turbulent blood flow through the stenosis coronary artery creates weak high-frequency murmurs, altering PCG waves. This provides a potential basis for PCG-based CAD detection. Nevertheless, when coronary stenosis exceeds 95%, the near-complete occlusion diminishes blood flow, resulting in the disappearance of turbulence and murmurs, which complicates the detection of severe CAD using PCG alone [6,7]. Single-modal detection methods suffer from information deficiencies that limit their effectiveness. In contrast, multi-modal data can provide complementary information from various data sources, improving CAD detection accuracy. Recently, Leasure et al. [8] combined ECG and coronary angiography to predict the presence, location, and severity of coronary lesions. Alizadehsani et al. [9] integrated demographics, symptoms, biochemical testing, ECG, and echocardiogram to identify stenosis in major coronary arteries. However, their data primarily originated from hospital settings, limiting the generalization of their techniques. Additionally, multi-modal methods have been developed in detecting other cardiovascular diseases. An improved D-S theory, fusing ECG and PCG signals, confirmed superior accuracy over single ECG or single PCG in cardiovascular disease identification [10]. Zarrabi et al. [11] predicted myocardial infarction based on a decision system integrating ECG, PCG, and clinical data, outperforming single-modal models. These advancements indicate the extensive potential of multi-modal data in disease detection. However, these studies only emphasize information of multi-modal data itself and overlook other effective information, such as coupling information, leading to low detection precisions.

In the process of CAD detection, various features have been developed for ECG or PCG classification [12,13,14,15,16,17]. Notably, nonlinear analysis has demonstrated significant advantages owing to the inherent nonlinear and nonstationary characteristics of ECG and PCG signals. Among nonlinear features, entropy is widely recognized as a key measure of signal complexity or irregularity and has been applied extensively across various disciplines [18]. Cherif et al. [19] used wavelet packet transformation (WPT) to analyze PCG signals and reconstructed different PCG components for computing entropy features and validating the effectiveness of entropy in PCG classification. Similarly, Liu et al. [20] used multi-channel PCG signals for CAD detection and confirmed that entropy features were more effective than other domain features. During various types of entropy, approximate entropy (ApEn), introduced by Pincus [21], has gained prominence in clinical cardiovascular studies [22], despite its limitations related to data length dependency and inconsistency. Sample entropy (SampEn) and fuzzy entropy (FuzzyEn) were further developed as an improvement over ApEn by optimizing its calculation [23,24]. However, SampEn and FuzzyEn require manual parameter setting, which introduces subjectivity and complicates standardization. In contrast, distribution entropy (DistEn), with its parameter independence, has demonstrated its superiority in the analysis of physiological signals [25]. However, entropy only provides valuable insights into the overall complexity of signals but ignores the details. Recurrence plot (RP) offers superior visualization of nonlinear dynamic changes of the signal and further enhances the analysis effect by capturing intricate details in combination with deep learning models [26,27].

**Figure 1:**
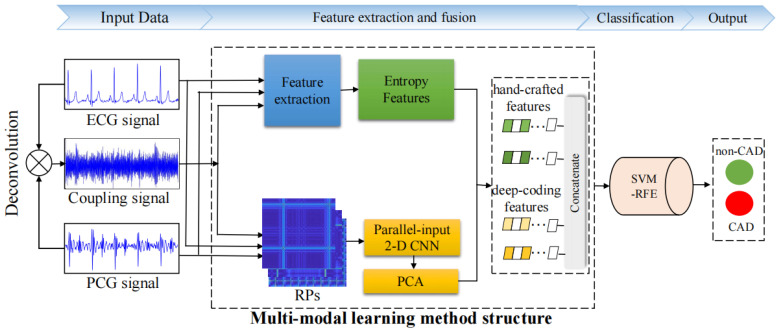
The block diagram of this proposed method.

Considering the above-mentioned reasons, this paper proposes a novel multi-modal learning method to integrate ECG, PCG, and their coupling signals for CAD detection, leveraging entropy and recurrence deep-coding feature measurements. The proposed method initially produces a novel coupling signal by operating deconvolution of ECG and PCG. Then, various entropy features are extracted from ECG, PCG, and their coupling signals to capture global information, followed by the use of recurrence deep-coding features to capture local details. A parallel-input 2-D CNN model was designed to encode deep features from RPs of multi-modal signals. After feature reduction and selection, an optimal multi-modal feature set was combined with support vector machine (SVM) for the final classification of CAD and non-CAD cases. The results demonstrate the efficacy of the multi-modal method and highlight the significant role of coupling features in improving CAD detection. Figure 1 presents the block diagram of CAD detection using the multi-modal method.

The contributions of this study to the literature are as follows:

- This study proposes a multi-modal learning method to enhance the precision of CAD detection by integrating ECG, PCG, and their novel coupling signals. The method initially generates a novel coupling signal based on the deconvolution of ECG and PCG, and then it extracts nonlinear features, including various entropy features and recurrence deep-coding features from multi-modal signals to capture global and local information.
- The proposed model designs a novel parallel-input 2-D CNN model to encode the deep representations of multi-modal RPs, enabling detailed analysis of cardiac state changes.

The rest of the sections of this study are organized as follows. Section 2 introduces data acquisition, data preprocessing, coupling signal evaluation, RP construction, CNN construction, feature reduction and selection, classification, and performance evaluation. Section 3 presents all the experimental results, and Section 4 compares and discusses the results. Section 5 concludes the study and outlines potential future research directions.

## 2. Materials and Methods

This study involved data collection from 199 recruited subjects at Shandong First Medical University Affiliated Hospital in Jinan, Shandong Province, China. This experiment was approved by the Ethical Review Committee of Shandong First Medical University Affiliated Hospital (ethics approval code: S374, approval date: 2020) and adhered strictly to the guidance of the Declaration of Helsinki and its amendments. All subjects presented with symptoms, such as chest tightness, chest pain, and palpitations over a week, and then provided informed consent before enrollment. The exclusion criteria were individuals who had undergone intervention of percutaneous coronary, coronary artery bypass surgery, or had been diagnosed as having valvular disease. The inclusion criteria were subjects who underwent coronary angiography, and diagnosis results were determined by the professional physician. Those with ≥50% blockages in at least one major coronary artery (left anterior descending, left circumflex, or right coronary artery) were diagnosed as CAD (135 positive cases), with the others as non-CAD (64 negative cases). Before the experiment, the basic information of subjects was as provided in Table 1, involving age, sex, height, weight, heart rate, and blood pressure.

To ensure accurate resting data collection, each participant was instructed to lie supine for at least 10 min in a quiet and controlled temperature (25 ± 3 °C) room before the experiment. A cardiovascular function detector (CVFD-II, Huiyironggong Technology Co., Ltd., Jinan, China) was applied to simultaneously record standard lead-II ECG and PCG signals for 5 min at a sampling rate of 1000 Hz. Subjects remained still and awake throughout the experiment.

### 2.1. Data Preprocessing

**Figure 2:**
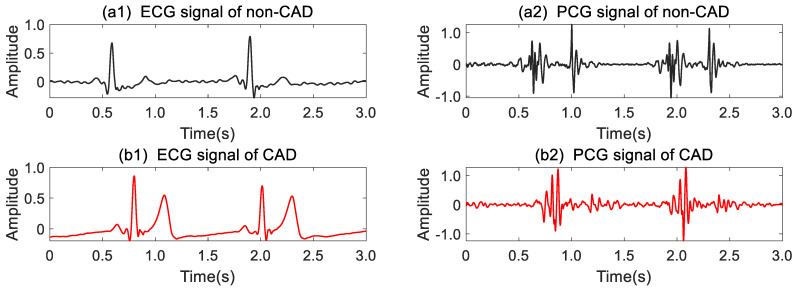
ECG and PCG signals of a non-CAD subject and a CAD patient after preprocessing.

During data collection, the raw signals were inevitably contaminated by various types of noises. To obtain clean ECG and PCG signals, a 0.5–60 Hz Butterworth passband filter and a 20 Hz high-pass Butterworth filter were employed to filter raw ECG and PCG signals, respectively. Subsequently, a 50 Hz IIR notch filter was designed to remove power frequency interference. In order to expand data, 5 min denoised ECG and PCG signals were cropped into 10 s segments, and we wholly obtained 199 × 30 sample segments of ECG and PCG, respectively. All segments were standardized using the z-score normalization for subsequent feature extraction. Figure 2 illustrates the preprocessed ECG and PCG segments from a non-CAD subject and a CAD patient, respectively. As shown in Figure 2(a1,b1), the CAD patient ST-waveform in ECG presented significant elevation compared to the gentle ST-wave in the non-CAD subject. Additionally, in the comparison of PCG in Figure 2(a2,b2), the non-CAD subject PCG showed distinct boundaries for the first heart sound (S1) and second heart sound (S2), whereas S2 of the CAD patient was nearly obscured with noises.

### 2.2. ECG–PCG Coupling Signal Evaluation

ECG and PCG signals provide valuable insights into cardiac electrical activity and mechanical activity, respectively. These two processes are closely interconnected through electromechanical coupling and mechanical-electrical feedback mechanisms [28]. Relying on coupling analysis between ECG and PCG, we can acquire the intrinsic relationship between these signals for reflecting complex dynamics of cardiovascular activities. It contains the amount of effective information over cardiovascular disease identification [29]. To leverage this correlation, we computed the deconvolution of ECG and PCG and produced a novel ECG–PCG coupling signal, further enhancing the understanding of cardiac function.

It is noted that cardiac electrical activity precedes mechanical activity. Based on this sequential process, we propose a novel electromechanical coupling model, where ECG serves as the input and PCG as the output, as defined in Equation (1). This model is designed to reflect the temporal and functional linkages between the two cardiac activities.

(1) y(n)=x(n)∗h(n)

Here, ∗ denotes convolution operation. *y*(*n*) denotes PCG signal, consisting of 2*N* − 1 sample points. *x*(*n*) is the ECG signal with *N* sample points, and *h*(*n*) is the novel ECG–PCG coupling signal with *N* sample points. Along with deconvolution calculation, the ECG–PCG coupling signal *h*(*n*) was evaluated successfully.

In the process of the deconvolution operation, *x*(*n*) and *h*(*n*) were padded with *N* − 1 zeros until the lengths of both signals reached 2*N* − 1. This preparation facilitated the transformation of the one-dimensional input signal *x*(*n*) into the matrix X with rank-*L*, thereby converting the convolution operation in Equation (1) into a more efficient matrix-vector calculation format in Equation (2).

(2) y=Xh

where X is rank-*L* convolution matrices of the form

(3) $$X=\left[\begin{array}{ccccc} x(0) & x(L-1) & x(L-2) & \cdots & x(1)\\ x(1) & x(0) & x(L-1) & \cdots & x(2)\\ x(2) & x(1) & x(0) & \cdots & x(3)\\ \vdots & \vdots & \vdots & \vdots & \vdots \\ x(L-1) & x(L-2) & x(L-3) & \cdots & x(0) \end{array}\right]$$

**Figure 3:**
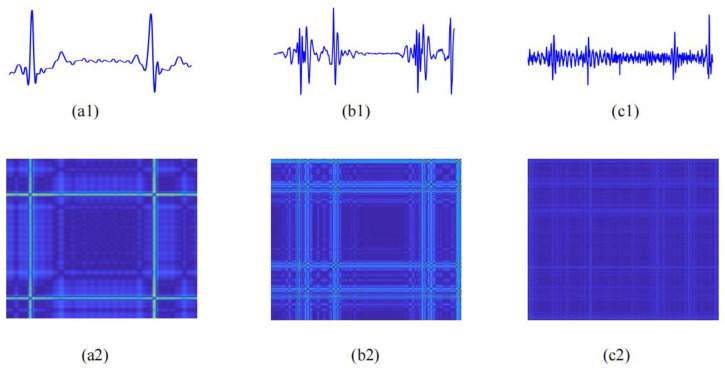
ECG, PCG, and ECG–PCG coupling signals and RPs of a non-CAD subject. (**a1**) ECG signal; (**a2**) RP of ECG signal; (**b1**) PCG signal; (**b2**) RP of PCG signal; (**c1**) ECG–PCG coupling signal; (**c2**) RP of ECG–PCG coupling signal.

**Figure 4:**
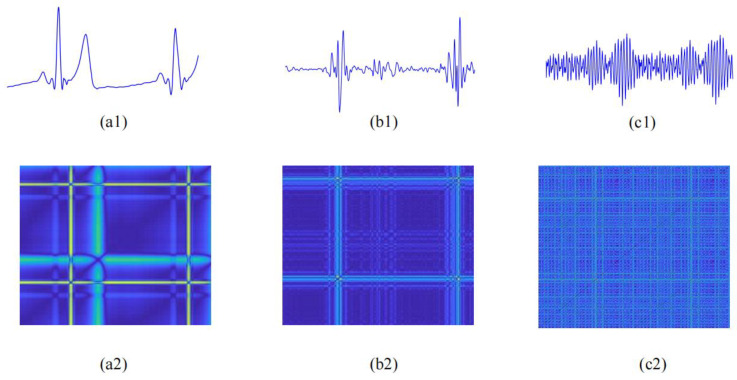
ECG, PCG, and ECG–PCG coupling signals and RPs of a CAD subject. (**a1**) ECG signal; (**a2**) RP of ECG signal; (**b1**) PCG signal; (**b2**) RP of PCG signal; (**c1**) ECG–PCG coupling signal; (**c2**) RP of ECG–PCG coupling signal.

Here, *L* is equal to 2*N* − 1. y is the column vector of output signal *y*(*n*), and h is the column vector containing electromechanical system parameters.

$$y=[{\begin{array}{ccccc} y(0) & y(1) & y(2) & \cdots & y(2N-1)] \end{array}}^{T}\;h=[{\begin{array}{ccccc} h(0) & h(1) & h(2) & \cdots & h(2N-1)] \end{array}}^{T}$$

where (.)*^T^* represents vector transposition. Within the operating matrix calculation in Equation (2), *h*(*n*) is evaluated successfully. Figure 3(c1) and Figure 4(c1) vividly display the ECG–PCG coupling signals of a non-CAD subject and a CAD patient, respectively. Notably, there were many significant differences between CAD and non-CAD subjects. In the ECG–PCG coupling signal, the non-CAD subject showed a lesser change in waves, whereas the CAD patient waves changed more obviously.

### 2.3. Feature Extraction

In automatic detection techniques, feature extraction is an essential step following data preprocessing. Given the inherent nonlinear and nonstationary characteristics of ECG and PCG signals, nonlinear analysis is particularly effective in capturing the dynamic variations within these signals. Therefore, this study adopted nonlinear analysis, specifically entropy and recurrence deep-coding measurements, to differentiate between CAD patients and non-CAD subjects using the multi-modal method.

#### 2.3.1. Entropy Features

Entropy measures the complexity and irregularity of time series data, providing valuable insight into the structure and dynamics of physiological signals. In this study, we extracted ApEn, SampEn, FuzzyEn, and DistEn features from all single-modal signals to analyze CAD.

1. ApEn [22] is a nonlinear analytical tool used to statistically quantify the regularity of the new signal patterns. Through phase space reconstruction and the new signal pattern generation, ApEn is calculated by comparing similar distances between all new patterns, which is defined as follows:
  (4) $$ApEn(m,r,N)=\frac{1}{\left(N-m+1\right)}(\sum_{i=1}^{N-m+1}\log C_{i}^{\left(m\right)}(r)-\sum_{i=1}^{N-m+1}\log C_{i}^{\left(m+1\right)}(r))$$
  where *N* represents the number of sample points in a signal, *m* denotes the embedding dimension, *r* is the threshold value, and *C* indicates the probability of similar distances between any two new patterns.

2. SampEn, an improvement algorithm of ApEn, overcomes the limitations of ApEn by excluding the probability of identical patterns [23]. It is also a prevalent tool used to measure the complexity of time series. It is calculated as follows:
  (5) $$S\mathrm{amp}En(m,r,N)=-\ln\frac{\sum_{i=1}^{N-m}B_{i}^{\left(m+1\right)}(r)}{\sum_{i=1}^{N-m}B_{i}^{\left(m\right)}(r)}$$
  where *N* denotes the number of sample points in signals, *m* is the embedding dimension, *r* is the threshold parameter, and *B* represents the probability of similar patterns.

3. FuzzyEn [30] introduces a fuzzy membership function to improve the SampEn algorithm. In the process of FuzzyEn calculation, the fuzzy similar distance *S_ij_* replaces the actual distance of the new patterns, which is defined as
  (6) $$S_{ij}^{m}=e^{\left(-d_{ij}^{2}/r\right)}$$
  where *d_ij_* denotes the distance between the new patterns. As was proven, incorporating the fuzzy membership function notably enhances FuzzyEn stability and consistency [31]. During the calculation of ApEn, SampEn, and FuzzyEn in this study, *m* was set to 2 and *r* was 0.2 times the standard deviation of the input signal [32].

4. DistEn [25] measures the complexity of the distance matrix by using empirical probability distribution functions (ePDF). Evaluation of ePDF mainly relies on the histogram of a predefined bin size *B*. It is defined by Shannon’s formula:

(7) $$DistEn(m)=-\frac{1}{\log_{2}(B)}\sum_{m=1}^{B}p_{t}\log_{2}(p_{t})$$

where *B* is set to 2ˆ8. The value of DistEn ranged from 0 to1 in this study.

According to Fourier analysis, it is evident that PCG exhibits a broad spectrum consisting of heart sound components and murmurs. For the analysis of the PCG signal, this study adopted wavelet pocket transform (WPT), utilizing a Daubechies-10 mother wavelet with a scale of 3 to decompose the PCG signal [19]. At level 3, 8 nodes spanning from (3, 0) to (3, 7) were obtained, each with a consistent frequency resolution of 62.25 Hz. To analyze the heart sound component with a primary frequency band of 10–200 Hz, we selected nodes (3, 0) to (3, 2) to reconstruct the signal with the components of S1 and S2, named as the S1-S2-signal (S1–S2s). Similarly, nodes (3, 3) to (3, 7) were selected to reconstruct the signal containing murmurs, defined as heart-murmur-signal (HMs). Subsequently, we extracted ApEn, SampEn, FuzzyEn, and DistEn from the 8 frequency sub-band; S1–S2s; HMs; PCG; ECG; and ECG–PCG coupling signals, yielding a total of 52 entropy features.

#### 2.3.2. Recurrence Deep-Coding Features

To characterize the local and microstructural nonlinearity of physiological signals, we constructed a recurrence plot (RP) to integrate the deep learning network based on its advantages in image feature extraction in order to encode deep feature representations of RP.

##### Recurrence Plot Construction

Entropy features statistically measure the complexity of the overall signal but overlook more detailed microstructural changes. To obtain these finer details, we transformed ECG, PCG, and ECG–PCG coupling signals into recurrence plots (PRs) based on phase space reconstruction to observe microstructural forms of cardiovascular status, providing valuable insights into the underlying dynamics. By reconstructing new patterns of these signals with time delay parameters τ = 1 and embedding dimension parameter m = 1 [33], RPs were constructed to quantify subtle variations in these signals.

Traditional RP relies on the threshold parameter to assign the grayscale values of recurrence points. However, an inappropriate threshold can result in the loss of crucial detailed information. To address this limitation and improve the visualization of dynamic details, we proposed a color-coded RP in a two-dimensional (2-D) image format. Rather than employing a fixed threshold, this method uses a color scale to represent the distances between phase space vectors. The distances between the vector at time i and all other vectors are mapped into a color spectrum, as defined in Equation (8), providing a more nuanced and detailed visualization of the signal dynamics.

(8) $$\upsilon \left(i,j\right)=\vartheta (\left\|\vec{X}(i)-\vec{X}(j)\right\|)$$

where ‖·‖ represents Euclidean distance, and υ denotes the color mapping function that assigns a distance value to a corresponding color within the spectrum. The color code for each pair of vectors *X*(*i*) and *X*(*j*) is positioned at the coordinate (*i*, *j*) in the color-coded RP, allowing for the quantification of additional nonlinear information in the physiological signal. In this visualization, a gradient of darkness indicates closer distances between vectors, while brighter colors represent larger distances. Figure 3 and Figure 4 illustrate RPs of ECG and PCG as well as coupling signals from non-CAD and CAD subjects, respectively. In comparison with the non-CAD subjects, the RP of the CAD patients exhibited more notable alterations compared to that of non-CAD individuals, highlighting the potential of RPs in identifying subtle differences related to CAD.

##### Parallel-Input CNN Framework

**Figure 5:**
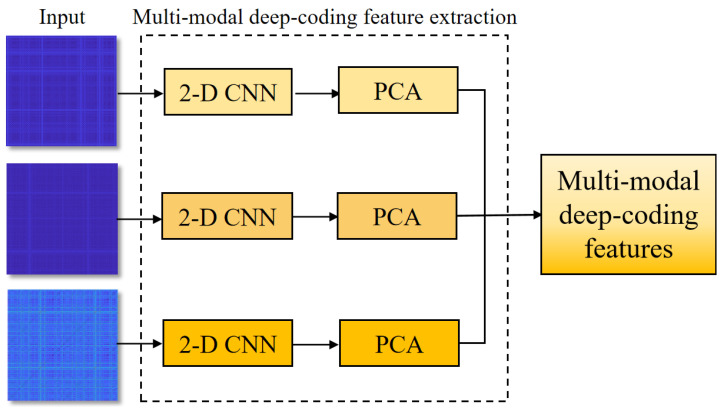
Structure of the parallel-input CNN framework.

The deep learning method has achieved great success across various fields, particularly in feature extraction and anomaly classification. CNN is adept at encoding spatial information of an image, offering significant advantages in image recognition tasks [34]. To effectively extract disease-related features for identifying CAD, this study designed a parallel-input CNN framework to encode deep feature representations from RPs of ECG, PCG, and ECG–PCG coupling, respectively. The architecture of this parallel-input CNN framework is illustrated in Figure 5.

**Figure 6:**
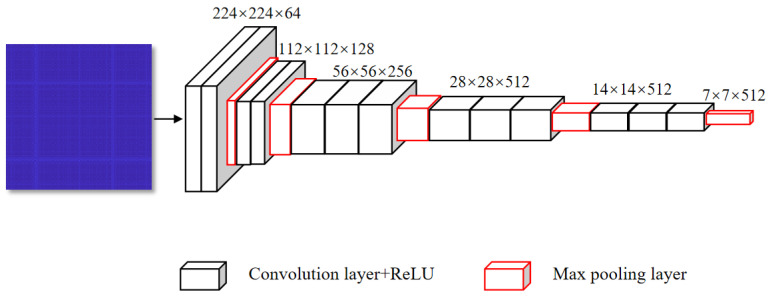
Architecture of the multilayer 2-D CNN network.

The proposed framework is based on the ensemble of three simple multilayer 2-D CNN models, each receiving one of the following inputs including RPs of ECG, PCG, and ECG–PCG coupling. The 2-D CNN model architecture is illustrated in Figure 6. Each 2-D CNN model was constructed to code deep features of the corresponding single-modal RP by resizing each RP to 224 × 224 × 3 as the input. The CNN model included 13 convolutional layers, each employing a 3 × 3 kernel to encode deep feature representations. These convolutional layers were partitioned into 5 sections, with 64, 128, 256, 512, and 512 convolutional kernels in each section, respectively. A maximum pooling layer based on 2 × 2 kernel followed the end of each convolutional section, responsible for the purpose of reducing feature image sizes and computational burden. All parameter configurations of convolutional and pooling layers are detailed in Table 2. In the proposed network, convolutional and pooling layers constituted the core modules for feature extraction. The output from each layer, referred to as deep features, carried rich spatial information from the RPs. All outputs were flattened and concatenated to form a multi-modal deep-coding feature set. The total amount of spatial image features relies on the number of kernels in each layer, as defined by Equations (9) and (10).

(9) $$y_{j}=\sum_{i}z_{ij}\times x_{i}+b_{i}$$

(10) $$y_{t}=\max(\sum_{n}z_{nt}\times x_{n})$$

where *x_i_* and *x_n_* denote the input image features as inputs of convolutional and max pooling layers, respectively; *y_j_* and *y_t_* are outputs of convolutional and max pooling layers, respectively; *z_ij_* and *z_nt_* are convolutional kernel and max pooling kernel, respectively; and *b_i_* is the bias of each layer for each layer.

The rectified linear unit (ReLU) serves as the activation function in all hidden layers. Additionally, different model parameters were investigated, and the optimal parameter was finally obtained. However, given the inherent complexity of the CNN structure, the outputs of the last pooling layer were flattened into the deep-coding feature set within a high-dimensional space. To mitigate the risk of overfitting associated with these high-dimensional feature vectors, we implemented the dimension reduction technique to enhance the generalization ability of the model.

### 2.4. Feature Reduction

This study utilized principal component analysis (PCA) to reduce the dimensionality of multi-modal deep-coding features, minimizing the risk of over-fitting. PCA transforms a high-dimensional feature vector, which may be correlated, into a lower-dimensional, uncorrelated feature vector through orthogonal projection, while preserving the essential information of the original vector. For a feature set ***X*** comprising *n* feature vectors with *m* dimensions, its covariance matrix ***C****_n_*_×*n*_ is initially computed as follows:

(11) $$C_{n\times n}=\sum_{i=1}^{m}x_{ni}x_{in},\;\;i=1,2,3,\cdots ,m$$

where *x_ij_* represents the elements of the feature set ***X***. After covariance matrix calculation, we further computed eigenvalues and eigenvectors of ***C****_n_*_×*n*_ in the projected space, as follows:

(12) $$C_{n\times n}w_{i}=\lambda_{i}w_{i}$$

where *λ_i_* and *w_i_* are the ith eigenvalue and corresponding eigenvector of ***C****_n_*_×*n*_, respectively.

The eigenvectors are then arranged in descending order based on the contribution rates of the corresponding eigenvalues. The eigenvector associated with the largest eigenvalue defines the first principal component, contributing most significantly to the representation of the original feature set. Principal components with higher contribution rates are retained to form a reduced feature set for CAD analysis. In this study, a cumulative contribution rate threshold of 90% was established to optimize the dimensionality of the ECG and PCG deep-coding feature sets.

### 2.5. Statistical Analysis

To assess whether the extracted features differed significantly between CAD and non-CAD subjects, a statistical analysis strategy was performed. The Kolmogorov–Smirnov test was used to check the normality of feature distributions, while Student’s *t*-test was employed for comparisons between non-CAD and CAD cases. For features with non-normal distributions, the Mann–Whitney U test was applied [35]. A significance level of *p* < 0.05 was established, indicating statistical differences of the extracted features, whereas a smaller *p*-value demotes a more significant statistical difference.

### 2.6. Classification

To enhance classification performance, this study employed the recursive feature elimination (RFE) algorithm to assess the importance score of each feature and sorted them accordingly for final classification tasks [36]. The selected optimal features were combined with SVM based on both linear kernel and radial basis function (RBF) kernel to identify CAD. The linear kernel incorporates a hyperparameter C, while RBF kernel utilizes two hyperparameters C and r. These optimal hyperparameter values were determined through a grid search with specified ranges: C ranging [2^−4^, 2^13^], and r with [2^−7^, 2^6^] [37], to overcome the potential overfitting impact and the classifier’s nonlinear behavior. This proposed method employs a 5-fold cross validation strategy to validate the model identification performance, ensuring the reliability of the classification results by deriving training and testing samples from different subjects.

### 2.7. Performance Evaluation

The final classification performance was assessed using a 5-fold cross-validation strategy, with the overall result being the average of five validation outcomes. To assess the classification performance, widely accepted metrics for anomaly classification were counted, including accuracy (*ACC*), sensitivity (*SEN*), specificity (*SPE*), and f1-score (*F1*).

(13) $$ACC=\frac{tp+tn}{tp+fp+tn+fn}\times 100\%$$

(14) $$SEN=\frac{tp}{tp+fn}\times 100\%$$

(15) $$SPE=\frac{tn}{tn+fp}\times 100\%$$

(16) $$F1=\frac{2tp}{2tp+fp+fn}\times 100\%$$

where *tp*, *tn*, *fp*, and *fn* represent true positive, true negative, false positive, and false negative samples, respectively.

## 3. Results

This study performed data preprocessing, coupling signal evaluation, feature extraction, RP construction, and statistical analysis using MATLAB R2020a (License No.: 40722615, Use: academic). The 2-D CNN network construction and classification were executed in open-source Python 3.9. The experiment was conducted on a PC with a 2.8 GHz Intel Core i5-8400 CPU and 8 GB RAM, running on Windows 10.

### 3.1. Feature Reduction Results

**Figure 7:**
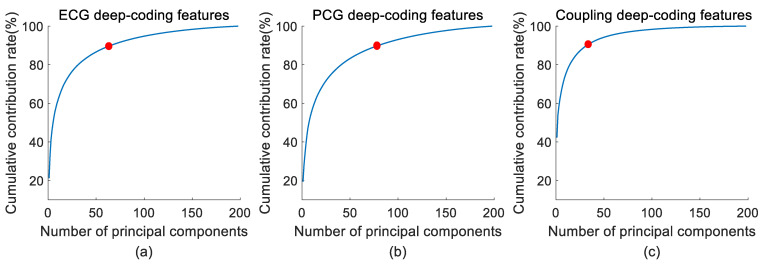
Recurrence deep-coding feature dimension reduction using PCA. (**a**) ECG deep-coding feature dimension reduction using PCA; (**b**) PCG deep-coding feature dimension reduction using PCA; (**c**) ECG–PCG deep-coding feature dimension reduction using PCA.

In addition to the various entropy extractions, this study also acquired the recurrence deep-coding features from ECG, PCG, and ECG–PCG coupling RPs by constructing the parallel-input 2-D CNN model. Due to the inherent complexity of the CNN architecture, we encoded the ECG deep-coding feature set, PCG deep-coding feature set, and coupling deep-coding feature set in high-dimensional space, potentially increasing the risk of over-fitting. To mitigate this concern, PCA was employed for feature reduction, which selected the principal components of the original ECG, PCG, and ECG–PCG coupling deep-coding feature set based on the cumulative contribution rate threshold of 90%. Figure 7 shows the cumulative contribution rates of ECG, PCG, and ECG–PCG coupling principal components. The cumulative contribution rate increased rapidly to 90%, and then the growth gradually declined. As a result, we selected the top 66 principal components from ECG deep-coding features, top 80 principal components from PCG deep-coding features, and top 35 principal components from ECG–PCG coupling deep-coding features. These selected deep-coding features, incorporated with 52 entropy features, were further used for CAD classification.

### 3.2. Statistical Analysis Results

**Figure 8:**
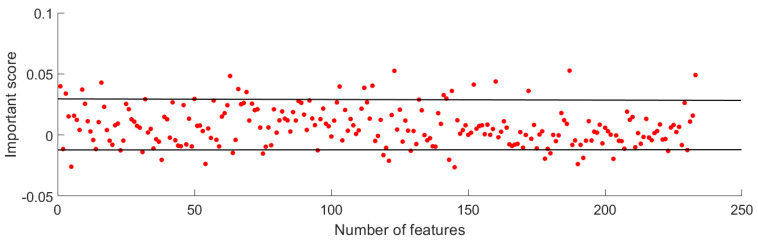
Important score of multi-modal features in classification using RFE.

Extracted entropy and recurrence deep-coding features provide a comprehensive representation of cardiovascular abnormalities, offering potential for CAD identification. Nevertheless, not all extracted features contribute equally to classification. The RFE algorithm was used to compute the important score of each feature in CAD classification, which is detailed in Figure 8. It can be seen that classification contribution rates of all features were located at an approximate range from −0.01 to 0.03, which indicates that the number of useful features with positive values exceeded the number of useless features with negative values. Since useless features have the opposite effect in detection, we ranked all extracted features based on important scores and further selected optimal features for classification.

After feature extraction and selection, statistical analysis was performed to evaluate the significance of all extracted features in MATLAB2020a (License No.: 40722615, Use: academic), and most effective features were selected for CAD detection. Table 3 illustrates the details of all features with statistically significant differences by using the t-test to compute *p*-values. A total of 42 features exhibited statistical differences, comprising 9 PCG deep-coding features, 8 ECG deep-coding features, 15 coupling deep-coding features, and 10 entropy features. Among these features, the 43rd principal component of ECG deep-coding features demonstrated the most significant statistical difference with the lowest *p*-value, highlighting the amount of impact that ECG deep-coding features have on statistical differences. The number of ECG–PCG coupling deep-coding features was largest, highlighting the critical role of coupling information in CAD detection. Additionally, entropy features also presented low *p*-values, denoting that entropy features exhibited more significant differences between non-CAD and CAD cases.

### 3.3. Classification Results

**Figure 9:**
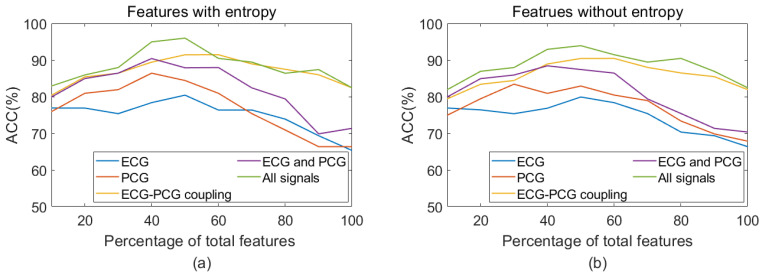
Classification accuracy of single-modal data and multi-modal data. (**a**) Accuracy of single-modal and multi-modal features with entropy; (**b**) Accuracy of single-modal and multi-modal features without entropy.

This study employed the RFE algorithm to select optimal features from the extracted multi-modal features for final classification. The selected optimal features as the input were fed into the SVM classifier to evaluate whether the integration of multi-modal features, as well as the inclusion of entropy features, enhances classification results. To assess the effect of multi-modal features on classification performance, a comparative analysis was conducted between single-modal and multi-modal features. All comparative results are shown in Figure 9. It notes that the multi-modal features consistently achieved superior performance over single-modal features within different percentages, either with or without entropy features.

**Figure 10:**
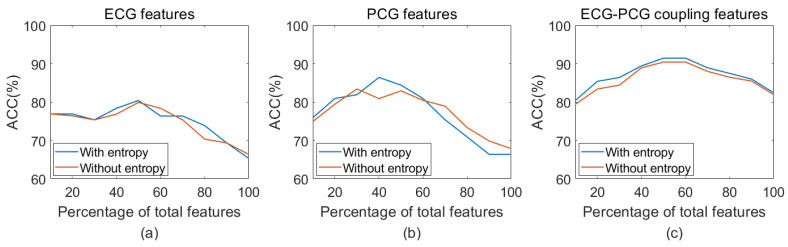
Classification accuracy using single-modal features with or without entropy features. (**a**) Accuracy of ECG features with or without entropy; (**b**) Accuracy of PCG features with or without entropy; (**c**) Accuracy of ECG–PCG coupling features with or without entropy.

**Figure 11:**
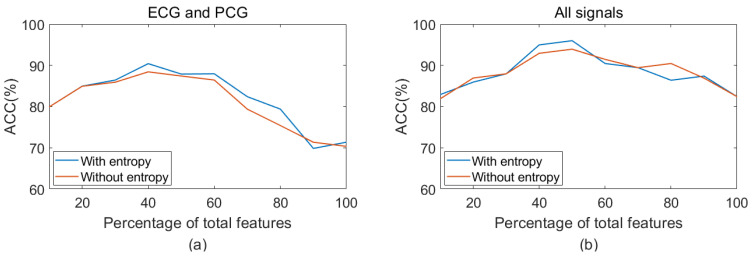
Classification accuracy using multi-modal features with or without entropy features. (**a**) Accuracy using integration of ECG and PCG features with or without entropy; (**b**) Accuracy using integration of ECG, PCG, and coupling features with or without entropy.

Additionally, this study further explored the impact of entropy features on classification performance, as illustrated in Figure 10 and Figure 11. For single ECG classification, the accuracy improved from 79.90% to 80.41% with the inclusion of entropy features. Similarly, in single PCG classification, accuracy increased from 84.41% to 86.41% following the introduction of entropy features. ECG–PCG coupling features obtained an increase from 90.42% to 91.44%, highlighting the significant contribution of coupling information in enhancing detection performance. When applying a combination of ECG and PCG, the higher accuracy of 90.42% was achieved, indicating performance improvement compared to single ECG or single PCG. The multi-modal method by integrating features of all signals yielded the best accuracy of 95.96%, increasing by almost 2% when entropy features were included. A comparative analysis indicated that both single-modal and multi-modal classification performance benefitted from the addition of entropy features.

Table 4 details a summary of the best classification results for single-modal and multi-modal features. After feature extraction, multi-modal features achieved the highest accuracy of 95.96%, which increased by 4.52% compared with the combination of ECG and PCG. Single-modal features also achieved the highest accuracies of 80.41%, 86.41%, and 91.44% in single ECG, PCG, and ECG–PCG coupling signals, respectively. In a comparison without entropy features, both single-modal and multi-modal features improved classification accuracy.

### 3.4. Classification Results of a Different Model

The complexity of the model can influence the detection accuracy. To confirm the superiority of the proposed method, we also compared a ResNet50-based model with deeper layers to our model. ResNet50 architecture includes the number of residual modules, which enhance feature extraction and reduce information loss. We adopted a ResNet50 structure instead of the 2-D CNN branch. Multi-modal signals and their MRPs were fed into the new model to extract entropy and deep-coding features for the classification. The detection performance of the ResNet50-based model is shown in Table 5. In comparison with our model, the ResNet50-based model acquires lower accuracy. It may indicate that the deeper model cannot capture more distinctive information about CAD in comparison with the shallow model.

## 4. Discussion

To alleviate the limitations associated with single-modal data in CAD detection, this study proposed a novel multi-modal learning method to integrate ECG, PCG, and its coupling signals. The proposed method adopted nonlinear analysis to extract various features, including entropy hand-crafted features and recurrence deep-coding features, for capturing global and local complex information, respectively. By leveraging the complementary strengths of multi-modal signals, we addressed the inherent limitations in single-modal method and attained remarkable improvements of CAD detection precision.

ECG waveforms exhibit pronounced changes in advanced CAD cases, making it relatively straightforward to distinguish severe CAD from non-CAD cases [5]. Nevertheless, subtle or absent symptoms in early or moderate CAD cases pose significant diagnostic challenges. Conversely, in patients with more than 20% coronary obstruction, turbulent blood flow leads to detectable changes in PCG waveforms, potentially aiding in CAD detection [6,7]. However, when coronary obstruction progresses to more than 95%, the reduction of blood flow and the disappearance of murmurs complicate the differentiation between severe CAD and non-CAD cases. This complexity underscores the limitations of single-modal signals and highlights the advantages of integrating multi-modal data, which provide a richer and more comprehensive view and improve detection accuracy.

In the context of CAD analysis utilizing a single-modal signal, ECG can easily identify severe CAD patients from non-CAD subjects, owing to significant ECG waveform alterations in the terminal stage, but it is difficult to identify early or moderate CAD patients. Conversely, significant PCG waveform changes in early or moderate CAD patients, while identifying severe CAD patients based on the absence of murmurs, becomes difficult. As a result, single ECG or single PCG provides insufficient information for CAD detection. In this study, non-CAD subjects were patients with less than 50% coronary obstruction. The existence of similar morphological changes between non-CAD and CAD cases brings difficulty in CAD detection, leading to lower classification accuracy, as shown in Figure 9 and Table 4. It is noted that PCG features yielded a high result in single-modal signals with an accuracy of 86.41%. The multi-modal method based on the integration of ECG and PCG signals achieved better improvement in classification. It indicates the presence of complementary information within different sources of data and demonstrates that multi-modal data effectively improve the detection performance of single-modal data. Therefore, integrating ECG and PCG features obtained the highest accuracy of 90.42%.

To further improve the detection precision, this study also considered intrinsic correlation information between ECG and PCG. The significant advantages of ECG–PCG coupling information in disease detection have been well proven [28,29]. Accordingly, we produced a novel coupling signal by operating the deconvolution of ECG and PCG, and then we extracted its entropy and deep-coding features for CAD analysis. The coupling information reflects an effective and intrinsic correlation between cardiac electrical and mechanical activities, thereby enhancing classification ability with a higher accuracy of 91.44% than single ECG or single PCG. Along with ECG and PCG features, we added coupling features, and the multi-modal method by integrating all signals yielded the best accuracy of 95.96%, significantly outperforming the combination of ECG and PCG. This indicates that the inclusion of multi-modal data provides more sufficient information for CAD detection, with the coupling information playing an important role in improving classification performance.

Nonlinear analysis has been proven to be advantageous in revealing dynamic characteristics of the signal due to its inherent nonlinearity. This study extracted entropy and recurrence deep-coding features to quantify ECG, PCG, and ECG–PCG coupling signals. To assess the validity of extracted features, statistical analysis was used to analyze statistical differences of each feature. The results showed that the ECG–PCG coupling deep-coding feature exhibited the most significant difference, while entropy features significantly contributed to overall classification ability, as indicated by the lower *p*-values in Table 3. By adding entropy features, both single-modal and multi-modal features achieved better improvement of the classification accuracy, as shown Figure 10 and Figure 11 and Table 4.

In terms of computational complexity and resource demands, the proposed model is relatively efficient and not overly time-consuming. The training process, encompassing feature extraction and classification, is completed in approximately 72 min, with each signal segment requiring about 22 s for training. After parameter optimization, the testing phase for each signal segment takes 13.52 s, 13.08 s for data preprocessing, entropy extraction, and RP construction, and 0.43 s for deep feature extraction and classification. In our model, initially extracted and reduced features are fused at the front end, and then they are used for optimal feature selection and classification. This design reduces memory resource requirements and accelerates the feature learning process, thereby enhancing the potential for real-time applications.

Table 6 provides an overview of existing research on CAD detection using single ECG or single PCG. Kaveh et al. [38] employed a fusion of multiple domain features from ECG for CAD classification and validated their model using the MIT-BIH ECG database. Samanta et al. [39], Li et al. [40], and Pathak et al. [41] focused on developing various PCG features for CAD detection using machine learning or deep learning techniques. Moreover, in multi-channel PCG signals, Liu et al. [20] employed multi-domain features for CAD detection, but achieved the accuracy of 90.92%. To address the challenge of data imbalance that affects generalization of the model, this study implemented an under-sampling strategy. Specifically, 64 CAD patients were randomly selected from the total CAD patients to balance the dataset, which was then used to retrain the classification model. The newly trained model, based on the new balanced dataset, demonstrated exceptional performance with an accuracy of 94.32%, sensitivity of 96.12%, and specificity of 93.44% in classifying 64 non-CAD and 64 CAD patients. After balancing data, we obtained similar accuracy but higher specificity than imbalanced data. To confirm the superiority of our model, we validated the multi-input CNN network developed by Li et al. [42] on our database, and we achieved the classification accuracy of 93.97%, which was slightly lower than our model. Moreover, the traditional model presented by Kumar et al. [12] was also validated on our database by extracting ECG and PCG time-frequency features and obtained the classification accuracy of 84.94%, significantly lower than our model.

In the field of CAD detection using single ECG or single PCG classification, numerous methods have been tested on the PhysioNet ECG database and the PhysioNet/CinC Challenge 2016 PCG database. To ensure a fair comparison with existing approaches, our multi-modal learning method was similarly validated on these two public databases, with details provided in Table 7. Previous studies on CAD detection using the public ECG database, such as those by Kumar et al. [12] and Acharya et al. [14], utilized hand-crafted features combined with machine learning techniques, while Tan et al. [13] employed time-domain deep-coding features through 1D-CNN. Recently, the use of PCG signals for CAD detection has gained attention, with most studies focusing on hand-crafted or deep-coding features [15,17]. Notably, Noman et al. [16] used MFCC deep features encoded by 2D-CNN. Given that the public databases only provide single-modal signals, our model, involving only single-modal features from public ECG and PCG databases, achieved high results with accuracies of 99.85% and 94.54%, respectively. These results highlighted the effectiveness and robust generalization capabilities of our model in CAD detection.

Nevertheless, this study has certain limitations. The data collection process was limited to only single ECG and single PCG recordings, missing the potential advantages of multi-modal integration. Incorporating additional data sources, such as imaging techniques or supplementary modalities, could substantially enhance the identification of CAD and improve classification performance. Additionally, the imbalance in data distribution across classes presents challenges in achieving optimal classification results. In future work, we will incorporate more additional modalities, such as images, physiological indicators, and multi-channel signals, to capture more sufficient information from various domains, thereby addressing existing limitations. We will also focus on data augmentation and expanding our dataset to address class imbalance and further improve the model performance. Furthermore, we will integrate the advantages of machine learning and deep learning approaches to develop a robust CAD detection system that provides valuable insights for healthcare professionals. Considering the complex management of coronary occlusion, we will further study coronary occlusion severity automatic detection for achieving early diagnosis and long-term detection. It is of great clinical significance to monitor and manage CAD.

## 5. Conclusions

Single-modal data, due to their inherent information limitations, can significantly affect the accuracy of CAD detection. To overcome these limitations, this study proposed a multi-modal learning method based on nonlinear analysis to integrate ECG, PCG, and ECG–PCG coupling signals for the purpose of improving CAD detection. The proposed method extracts nonlinear features, including entropy and recurrence deep-coding features, for analyzing CAD. After feature selection, optimal features are placed into the SVM for the final classification of non-CAD and CAD cases. The experimental results demonstrated that the proposed multi-modal method by integrating all signals achieved satisfactory detection performance with a high accuracy of 95.96%, significantly surpassing that of a single-modal signal and combined ECG and PCG. Additionally, the inclusion of entropy features, both in single-modal and multi-modal features, also effectively improves classification performance. This underscores advantages of the multi-modal method in providing more sufficient information for CAD detection, with ECG–PCG coupling features playing a crucial role in enhancing classification accuracy.

## Figures and Tables

**Table 1:** Basic information of all subjects (mean ± SD).

Characteristics	Non-CAD	CAD
Age	61 ± 10	62 ± 10
Male/female	30/34	89/46
Height	164 ± 7	166 ± 8
Weight	69 ± 12	71 ± 11
Heart rate	72 ± 12	75 ± 16
Systolic blood pressure	134 ± 15	133 ± 16
Diastolic blood pressure	80 ± 11	82 ± 12

**Table 2:** All detailed parameters of the multilayer CNN network.

Index	Layer	Index	Layer
1	conv3_64	10	max-pooling_2
2	conv3_64	11	conv3_512
3	max-pooling_2	12	conv3_512
4	conv3_128	13	conv3_512
5	conv3_128	14	max-pooling_2
6	max-pooling_2	15	conv3_512
7	conv3_256	16	conv3_512
8	conv3_256	17	conv3_512
9	conv3_256	18	max-pooling_2

Note: “conv (kernel size)_(number of kernels)” represents the convolutional size and the number, while “max-pooling_(kernel size)” represents the max-pooling layer parameters.

**Table 3:** Details of multi-modal features with statistical differences.

Feature	Type	*p*-Value	Feature	Type	*p*-Value
RD-pcg23	Deep-coding	0.0384	RD-coupl22	Deep-coding	0.0470
RD-pcg32	Deep-coding	0.0491	RD-coup23	Deep-coding	0.0418
RD-pcg40	Deep-coding	0.0431	RD-coup26	Deep-coding	0.0427
RD-pcg46	Deep-coding	0.0134	RD-coup27	Deep-coding	0.0444
RD-pcg51	Deep-coding	0.0028	RD-coupl29	Deep-coding	0.0335
RD-pcg57	Deep-coding	0.0082	RD-coupl30	Deep-coding	2.54 × 10^−5^
RD-pcg58	Deep-coding	0.0271	RD-coupl31	Deep-coding	0.0421
RD-pcg69	Deep-coding	0.0117	RD-coupl32	Deep-coding	0.0311
RD-pcg76	Deep-coding	0.0318	RD-coupl33	Deep-coding	0.0014
RD-ecg9	Deep-coding	0.0268	RD-coupl34	Deep-coding	0.0311
RD-ecg17	Deep-coding	0.0037	RD-coupl35	Deep-coding	0.0014
RD-ecg21	Deep-coding	0.0022	ApEn-pcg-1	Entropy	2.39 × 10^−4^
RD-ecg29	Deep-coding	0.0176	SampEn-pcg-1	Entropy	2.38 × 10^−4^
RD-ecg36	Deep-coding	0.0174	FuzzyEn-pcg-1	Entropy	2.41 × 10^−4^
RD-ecg43	Deep-coding	4.66 × 10^−7^	DistEn-pcg-1	Entropy	2.39 × 10^−4^
RD-ecg63	Deep-coding	0.0142	ApEn-s1-s2s	Entropy	0.0295
RD-ecg64	Deep-coding	0.0418	SampEn-pcg	Entropy	0.0194
RD-coupl13	Deep-coding	0.0478	FuzzyEn-pcg	Entropy	0.0298
RD-coupl15	Deep-coding	0.0307	DistEn-pcg	Entropy	0.0433
RD-coupl16	Deep-coding	0.0199	SampEn-ecg	Entropy	0.032
RD-coupl20	Deep-coding	0.023	SampEn-coupl	Entropy	0.032

Note: ‘RD-pcg*n*, RD-ecg*n*, and RD-coupl*n*’ denotes the *n*th principal component of ECG, PCG, and ECG–PCG coupling recurrence deep-coding features, respectively. ‘En-pcg-*n*’ denotes the entropy feature of *n* node in the PCG signal. ‘En-*x*’ is the different entropy features of single-modal signal *x*.

**Table 4:** The best classification results of single-modal and multi-modal features.

	With Entropy				Without Entropy			
	ACC (%)	SEN (%)	SPE (%)	F1 (%)	ACC (%)	SEN (%)	SPE (%)	F1 (%)
Single ECG	80.41 ± 2.85	89.63 ± 3.63	61.15 ± 12.42	66.07 ± 8.10	79.90 ± 3.54	90.37 ± 2.96	57.95 ± 9.94	64.58 ± 7.67
Single PCG	86.41 ± 4.15	96.29 ± 4.68	65.64 ± 14.19	74.85 ± 9.27	84.41 ± 4.34	92.59 ± 3.31	67.18 ± 8.94	73.30 ± 7.93
Single coupling	91.44 ± 2.56	94.81 ± 5.54	84.48 ± 4.67	86.54 ± 3.40	90.42 ± 3.44	95.56 ± 2.77	79.49 ± 9.86	83.89 ± 6.59
ECG and PCG	90.42 ± 3.45	95.56 ± 2.77	79.36 ± 9.32	79.97 ± 5.99	89.42 ± 3.04	93.33 ± 4.32	81.03 ± 9.21	82.74 ± 5.97
All signals	95.96 ± 3.05	100.00 ± 0.00	87.31 ± 9.67	92.93 ± 5.60	93.97 ± 4.89	98.52 ± 1.81	84.36 ± 1.61	89.10 ± 9.74

Note: results are the average and standard deviation of five validations (mean ± SD).

**Table 5:** Classification results of different models.

Model	ACC (%)	SEN (%)	SPE (%)	F1 (%)
ResNet50-based model	90.96 ± 2.89	94.81 ± 1.81	82.82 ± 5.71	85.45 ± 4.80
Our model	95.96 ± 3.05	100.00 ± 0.00	87.31 ± 9.67	92.93 ± 5.60

**Table 6:** Summary of existing studies on the diagnosis of CAD.

Author	Data	Method	Result (%)
Liu et al. [20]	Self-collected 21CAD/15non-CAD	Multi-channel PCG; time domain, frequency domain, and nonlinear domain features; SVM	ACC:90.9 SPE:93.0 SEN:87.9
Kaveh et al. [38]	MIT-BIH 43CAD/46non-CAD	ECG; time domain and frequency domain features; SVM	ACC:88.0 SPE:92.6 SEN:84.2
Samanta et al. [39]	Self-collected 29CAD/37non-CAD	PCG; time domain and frequency domain features; CNN	ACC:82.6 SPE:79.6 SEN:85.6
Li et al. [40]	Self-collected 135CAD/60non-CAD	PCG; multi-domain features; deep features; MLP	ACC:90.4 SPE:83.4 SEN:93.7
Pathak et al. [41]	Self-collected 40 Normal/40 CAD	PCG; imaginary part of cross power spectral density; SVM	ACC: 75.0 SPE: 73.5 SEN: 76.5
This study	Self-collected 135CAD/64non-CAD	ECG and PCG; entropy; RP; deep learning and SVM	ACC:95.96 SPE:87.43 SEN: 100.00
This study	Self-collected 64CAD/64non-CAD	ECG and PCG; entropy; RP; deep learning and SVM	ACC:94.32 SPE: 93.44 SEN: 96.12

**Table 7:** Comparison of existing studies on ECG classification using the PhysioNet dataset and PCG classification using the PhysioNet/CinC Challenge 2016 dataset.

Author	Classifier	Input	Result (%)
Studies on ECG classification using the PhysioNet dataset			
Kumar et al. [12]	SVM	Time-frequency features	ACC: 99.60
Tan et al. [13]	1-D CNN	ECG signal	ACC:99.85
Acharya et al. [14]	1-D CNN	Entropy features	ACC:99.27
This study	SVM	Entropy, recurrence deep-coding features	ACC: 99.85
Studies on PCG classification using the PhysioNet/CinC Challenge 2016 dataset			
Tschannen et al. [15]	1-D CNN	Time features, frequency features	ACC: 87.00
Noman et al. [16]	2-D CNN	MFCCs image	ACC: 88.80
Baydoun et al. [17]	Boosting and bagging model	Time-frequency features, statistical features	ACC: 91.50
This study	SVM	Entropy, recurrence deep-coding features	ACC: 94.54

## Data Availability

The data are available on request.
